# Improvement of Post-Surgery Constipation in Patients with Fractures by *Lactobacillus rhamnosus* JYLR-127: A Single-Blind Randomized Controlled Trial

**DOI:** 10.3390/nu16101505

**Published:** 2024-05-16

**Authors:** Yiyang Han, Yunlong Zhou, Xuan Xu, Shen Chen, Shuwei Zhang, Nan Jiang, Zhiqiang Liu, Junyu Zhang, Zhaowei Luo, Xinfeng Zhang, Liang Hao, Tingtao Chen

**Affiliations:** 1 Department of Orthopedics, The 2nd Affiliated Hospital, Jiangxi Medical College, Nanchang University, Nanchang 330006, China; hyydry@outlook.com; 2 Queen Mary School, Jiangxi Medical College, Nanchang University, Nanchang 330031, China; shensen0709@163.com (S.C.); zhangshuwei0506@163.com (S.Z.); n.jiang20@outlook.com (N.J.); 3 Department of Orthopedics, Leshan People’s Hospital, Leshan 614003, China; doctorzyl42696780@126.com (Y.Z.); spinelzq@163.com (Z.L.); doczjy@163.com (J.Z.); 4 Institute of Translational Medicine, Jiangxi Medical College, Nanchang University, Nanchang 330031, China; xuxuan040618@126.com (X.X.); lzw48hours@163.com (Z.L.); 5 School of Pharmacy, Jiangxi Medical College, Nanchang University, Nanchang 330031, China; zxf55623@sina.com

**Keywords:** constipation after fracture surgery, probiotic preparation, *Lactobacillus rhamnosus*, adjuvant therapy, gut microbiota

## Abstract

The high prevalence of constipation after fracture surgery brings intolerable discomfort to patients on the one hand, and affects post-surgery nutrient absorption on the other hand, resulting in poor prognosis. Given the acknowledged probiotic properties of *Lactobacillus rhamnosus*, 100 fracture patients with post-surgery constipation were centrally enrolled and administered orally with *L. rhamnosus* JYLR-127 to assess the efficacy of probiotic-adjuvant therapy in alleviating post-fracture constipation symptoms. The results showed that *L. rhamnosus* JYLR-127 improved fecal properties, promoted gastrointestinal recovery, and relieved constipation symptoms, which were mainly achieved by elevating Firmicutes (*p* < 0.01) and descending Bacteroidetes (*p* < 0.001), hence remodeling the disrupted intestinal microecology. In addition, blood routine presented a decrease in C-reactive protein levels (*p* < 0.05) and an increase in platelet counts (*p* < 0.05) after probiotic supplementation, prompting the feasibility of *L. rhamnosus* JYLR-127 in anti-inflammation, anti-infection and hemorrhagic tendency prevention after fracture surgery. Our study to apply probiotics in ameliorating constipation after fracture surgery is expected to bless the bothered patients, and provide broader application scenarios for *L. rhamnosus* preparations.

## 1. Introduction

Bone fracture is defined as a disruption of the integrity or interruption of the continuity of any bone in the body, with deformity, dyskinesis, and bony crepitus as the exclusive signs [1]. The latest statistics released by *Lancet Healthy Longev* put the new fractures at 178 million worldwide in 2019, while the prevalent cases of existing acute or chronic fracture symptoms reached a staggering 455 million [2]. Most fractures are attributed to direct or indirect violence and are complicated by bacteremia, fat embolism, or shock, leading to disability and even mortality in severe trauma [3,4]. Acute phase fracture treatment is dominated by fixation with plaster and, in some cases, endo-skeletal repositioning [5]. However, the ensuing prolonged bed rest and limited mobility after surgery inevitably result in adverse reactions such as constipation, anorexia and lassitude [6]. Thus, searching for an adjunctive therapy or preparation that helps ameliorate symptoms like constipation in the post-surgery rehabilitation of fracture patients may be a preferred solution.

As a subjective complication of various diseases, constipation mainly presents as deficient or difficult defecation, accompanied by abdominal pain and bloating [7]. In patients with fractures, constipation can occur during post-surgery recovery from multiple fracture sites, including the thoracolumbar spine, hip, femur, and ribs [8]. The static state of the body after surgery will cause a weakening of intestinal peristalsis function, coupled with dietary factors, thereby inducing constipation, whose incidence is generally 50–70% [9]. At current, mounting research has accentuated the correlation between gut microbiota and constipation. In a clinical trial among 70 elderly patients with gastrointestinal dysfunction after hip fracture repositioning, Liang et al. [10] found that the test group showed less *Bifidobacterium* and *Lactobacillus* than healthy controls before intervention, whereas *Enterobacterium* and *Enterococcus* was the opposite. After treatment with the Chinese herbal decoction, a reversed abundance of the above bacteria and improved constipation scores were observed. Another randomized controlled study in patients with post-surgery constipation after thoracolumbar fractures displayed an analogous trend [11]. Hence, gut microbiota modulation may be a potential target for alleviating constipation symptoms in post-surgery fracture rehabilitation.

Probiotics are living microorganisms that benefit host health when consumed in sufficient quantities [12]. By interacting with the organism and the environment through their own bacterial components or derived metabolites, probiotics function in the treatment and prognosis of gastrointestinal dysfunction, inflammation, and metabolic diseases [13]. Among them, *Lactobacillus rhamnosus*, one of the intestinal colonizing bacteria, possesses evident superiority in maintaining the gut microbiota balance, as well as certain therapeutic effects in strengthening immunity and anti-inflammation [14]. On the one hand, Lai et al. [15] reported that *L. rhamnosus* HN001 supplementation accelerated defecation frequency and relieved defecation tension in people with functional constipation. On the other hand, *L. rhamnosus* GG administration significantly reduced the systemic inflammatory level in rats with femoral diaphyseal fracture by remodeling dysbiosis, which was speculated to hold feasibility for the management of skeletal injuries and possible complications [16]. However, there are no relevant studies to further explore the specific efficacy of *L. rhamnosus* on post-fracture constipation to date.

On this basis, our study included 100 fracture patients with post-surgery constipation symptoms altogether and adopted *L. rhamnosus* JYLR-127 as an adjuvant intervention. 16S rDNA high-throughput sequencing and metabolomic analysis were performed to evaluate the effect of *L. rhamnosus* JYLR-127 on the post-surgery rehabilitation of fracture patients as well as the alleviation of associated constipation symptoms. The implementation of the trial is anticipated to provide microbial prognostic data support for the application of probiotics in the treatment of post-surgery constipation in patients with fracture, and at the same time pave the way for the industrialization of *L. rhamnosus*.

## 2. Materials and Methods

### 2.1. Ethical Statement

This study was a single-center, single-blind, parallel randomized controlled clinical trial in accordance with accepted ethical standards. Our project has been approved by the Ethics Committee of Leshan People’s Hospital (Leshan, China, AF/SC-04), and has also completed registration with the China Clinical Trial Registry (ChiCTR2300072781).

### 2.2. Patient Enrollment and Probiotics Preparation

From August 2022 to June 2023, the recruitment of constipation patients during post-surgery recovery from fractures was launched on the platform of Leshan People’s Hospital. Two expert clinicians performed routine medical examinations and professional assessments of all intended patients, including history taking, scale scoring, physical examination and blood tests. To obtain the ideal study subjects, specific inclusion and exclusion criteria were devised as follows:

Patients who met the following conditions were included: (1) aged 18–70, irrespective of gender; (2) fracture diagnosis confirmed by X-ray/CT images and managed with appropriate fixation or reduction; (3) symptoms of constipation, quantified by a score of ≥6 on the Chronic Constipation Severity Scale (CSS) and a score of <3 for fecal type on the Bristol Stool Scale (BSS), during the post-surgery rehabilitation phase; (4) were still in fracture post-surgery rehabilitation within one week of receiving probiotic intervention. Before enrollment, participants thoroughly overviewed the procedures, risks and precautions of the study and signed an informed consent form.

Participants with any of the below characteristics were excluded: (1) history of chronic constipation or uncertain about whether constipation symptoms were triggered by the fracture event; (2) have taken probiotic preparations (tablets, powders, capsules) within 2 weeks before the trial; (3) history of laxative medication usage within a month before the trial; (4) allergy or intolerance to probiotics or other ingredients; (5) previous history of abdominal and/or intestinal surgery, diagnosis of irritable bowel syndrome (IBS) or functional abdominal pain syndrome by Rome IV; (6) doubt about the capacity of subjects to complete the trial; (7) currently participating in another ongoing research. Unenrolled patients have also continued appropriate clinical healthcare as prescribed.

The probiotic powder and placebo involved in this study were manufactured by Shandong Zhongke Jiayi Bioengineering Co., Ltd. (Weifang, China) at 2 g per bag. The placebo was biologically inactive isomalto-oligosaccharide, while the probiotic powder was prepared by the low-temperature freeze-drying process and subsequently mixed into isomalto-oligosaccharide, at least 2 × 10^9^ colony-forming units (CFU) of *L. rhamnosus* JYLR-127 (CGMCC No. 24340) in each bag was guaranteed. When brewed in warm water at 35–40 °C, the probiotics can be evenly and stably distributed herein, with the texture and flavor almost identical to the placebo.

### 2.3. Trial Procedure

The trial was conducted at the Leshan People’s Hospital, where a dedicated staff was responsible for numbering and administering the participants presented with constipation symptoms during post-surgery fracture recovery. Prior to the commencement of intervention, half the patients were selected through computer-generated random number code to constitute the pre-intervention group (FAC group), scored for constipation using the Bristol Stool Fecal Characteristics Scale (BSFS), and stool samples were collected for subsequent experiments. The same method was applied to randomly assign the participants into the placebo group (CDN group) and the probiotic group (TRE group) on average. The documents stating the parameters, such as the blinding codes for the seeds, the block length, and the random numbers were sealed in envelopes and stored at the Institute of Translational Medicine, Nanchang University.

During the intervention, the TRE group received probiotics (*L. rhamnosus* JYLR-127) for one week at 4 g/day, twice daily (bid), 2 g/time, and the CDN group took placebo for one week accordingly at the same dose. At the endpoint, fecal samples were collected from all patients and the blood routine was measured again. The scores of the Bristol Fecal Characteristics Scale (BSFS) and Patient Assessment of Constipation Symptoms (PAC-SYM) were counted as outcome indicators for the trial.

### 2.4. Sample Collection

Blood routine and biochemical parameters were determined by blood sampling from the median cubital vein, collected in EDTA tubes and monitored by a hematology analyzer (Mindary BC6900, Mindary Biomedical Electronics Co., Ltd., Shenzhen, China). The analyzed indicators contained counts of white blood cells (WBC, 10^9^/L), red blood cells (RBC, 10^12^/L), platelet (PLT, 10^9^/L) and C-reactive protein (mg/L). Samples were collected from the interior of the middle and posterior sections of the feces into 50 mL EP tubes, and stored in a −40 °C freezer immediately after sampling.

### 2.5. High-Throughput Sequencing

Total DNA of fecal microorganisms was extracted from patients in the FAC, CDN and TRE groups. DNA was obtained using a genomic DNA kit (OMEGA Soil DNA Kit (D5625-01) (Omega Bio-Tek, Norcross, GA, USA) supplemented with bead-blast method. The concentration of genomic DNA and the purity of the samples were quantified by spectrophotometry (NanoDrop 2000, Thermo Fisher Scientific (China) Co., Ltd., Shanghai, China). The V4 region of 16S rRNA from each sample was amplified by the 338F/806R primers (338F, 5′-AYTGGYDTAAAGNG-3′; 806R, 5′-TACNVGGGTATCTAATCC-3′). PCR was performed under initial denaturation at 98 °C for 2 min, denaturation at 98 °C for 15 s, annealing at 55 °C for 30 s, extension at 72 °C for 30 s, and final extension at 72 °C for 5 min, maintained for 25–30 cycles at 10 °C. PCR products were sequenced by the Illumina NovaSeq platform. The raw reads were saved in FASTQ format and have been uploaded to the SRA database in NCBI (Bioproject No: PRJNA1093356).

### 2.6. Data Analysis

The sequencing results were denoised according to the QIME2 (version 2019.4) DADA2 analysis process, and the above-obtained sequences were merged by 100% sequence similarity to generate amplicon sequencing variants (ASVs) as well as abundance data tables. A Venn diagram was drawn to compare the richness and homogeneity of ASVs among samples. β-diversity analyses used UniFrac distance measurements in QIME2 software to investigate the microbial structural changes among samples, visualized by principal coordinate analysis (PCoA) based on genus-level species composition profiles. Microbial community characteristics that were markedly different and biologically significant in each group were also obtained by unweighted pair-group method with arithmetic means (UPGMA) clustering and visualized in the form of a heatmap. To guarantee comparability, the microbial sample data presented were normalized prior to analysis.

The data were analyzed using SPSS software (IBM SPSS, version 20.0) and GraphPad Prism software (version 9.0). Given that the sample data were all quantitative, satisfied normal distribution and were unpaired, statistical variability among the three groups was determined primarily through one-way analysis of variance (ANOVA). The method of multiple tests and *p*-value correction for differences among groups depended on the results of the variance chi-square test. The least significant difference (LSD) method was chosen when the variance was homogeneous, whilst the Tamhane method was adopted in the opposite. All data were presented as mean ± standard deviation (SD) and the significance level was set at *p* < 0.05. Pearson correlation analysis was used to calculate the correlation between the bivariate variables, with r values denoting positive/negative correlation as well as the magnitude of the correlation, and *p* < 0.05 indicating that the correlation between variables was statistically significant.

## 3. Results

### 3.1. Baseline Characteristics of Participants

**Figure 1:**
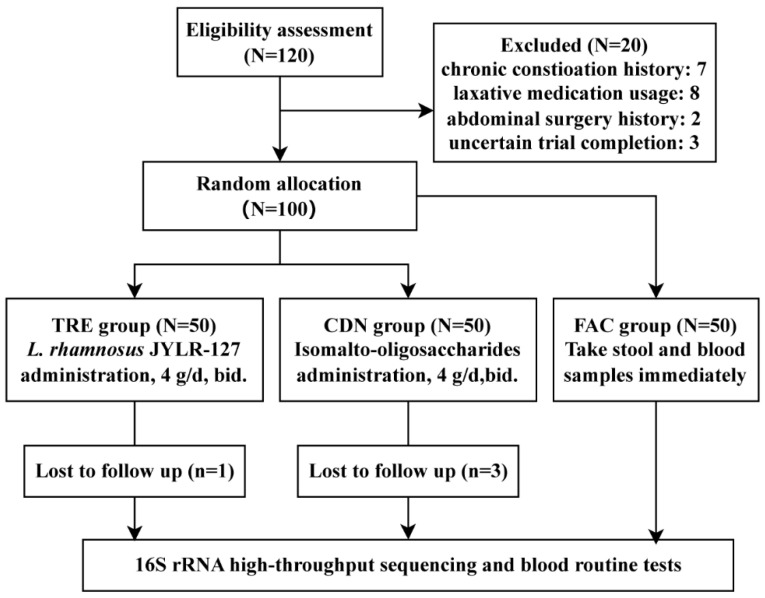
Experimental flowchart.

After stratified screening, 100 fracture patients with post-surgery constipation who met expectations were enrolled; the experimental layout is shown in Figure 1. Due to certain uncontrollable factors, four participants were lost to follow-up throughout the trial, forming an ultimate category of the FAC, CDN and TRE groups with 50, 47 and 49 subjects each. Baseline characteristics such as age and body mass index (BMI) showed no statistical difference among the three groups (FAC vs. CDN vs. TRE = 54.00 ± 11.08 vs. 53.85 ± 11.46 vs. 55.06 ± 10.14, *p* = 0.837; FAC vs. CDN vs. TRE = 22.30 ± 1.62 vs. 22.20 ± 1.61 vs. 22.04 ± 1.57, *p* = 0.979), and the proportions of gender and fracture type (according to fracture site) were also approximate, suggesting that the sample data were basically normal-distributed and comparable among groups (Table 1).

### 3.2. L. rhamnosus JYLR-127 Alleviated Post-Surgery Constipation and Reduced Inflammatory Response in Patients with Fracture

**Figure 2:**
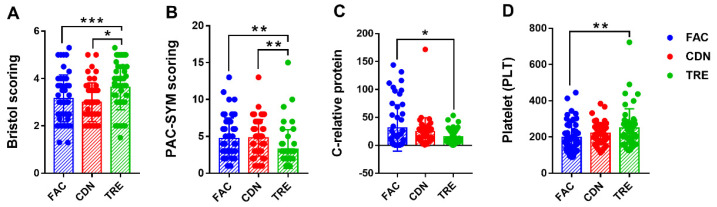
*L. rhamnosus* JYLR-127 improved post-surgery constipation symptoms and mediated anti-inflammation and anti-infection in the organism. (**A**) Bristol scoring; (**B**) PAC-SYM scoring; (**C**) C-relative protein concentration in blood routine; (**D**) PLT counting in blood routine. FAC: pre-intervention group (N = 50); CDN: placebo control group (N = 47); TRE: *L. rhamnosus* JYLR-127 group (N = 49). * *p* < 0.05, ** *p* < 0.01, *** *p* < 0.001.

The statistical results of fecal and blood indicators in the pre-intervention FAC group, and post-intervention CDN and TRE groups were given in Table 2. At the defecation level, the mean status in defecation, fecal traits (Bristol scoring) and constipation symptoms (PAC-SYM scoring) among groups and differences in between were recorded, respectively. As expected, the results of the one-way ANOVA presented significant intergroup differences in the above metrics. The defecation frequency was significantly higher in the *L. rhamnosus* JYLR-127-administered TRE group compared to the pre-intervention FAC group and the placebo-controlled CDN group (FAC vs. CDN vs. TRE = 2.24 ± 0.72 vs. 2.06 ± 0.70 vs. 3.04 ± 1.54, *p* < 0.001), with Bristol scoring closer to the median value of 3.50 (FAC vs. CDN vs. TRE = 3.16 ± 0.99 vs. 2.99 ± 0.82 vs. 3.63 ± 0.95, *p* < 0.01, Figure 2A). Meanwhile, the scores of rectal, abdominal, and fecal symptoms in PAC-SYM scoring of the TRE group decreased (Figure 2B), especially the improvement of abdominal symptoms showed obvious statistical differences over the other two groups (FAC vs. CDN vs. TRE = 1.04 ± 0.81 vs. 1.13 ± 0.82 vs. 0.41 ± 0.54, *p* < 0.001), which all implied the alleviating and ameliorating effect of *L. rhamnosus* JYLR-127 on the post-surgery constipation symptoms of fracture patients.

In addition, the potential involvement of *L. rhamnosus* JYLR-127 in diminishing inflammation in fracture patients with post-surgery constipation was assessed by measuring the variations in routine blood as well as blood biochemistry among the three groups. No obvious differences were seen in C-reactive protein concentration, erythrocyte and leukocyte counts within the three groups, and in particular, the levels of the latter two were almost equal, with statistical differences solely occurring in C-reactive protein concentration (FAC vs. CDN vs. TRE = 31.13 ± 41.58 vs. 24.45 ± 25.13 vs. 15.42 ± 13.02, *p* = 0.030, Figure 2C). Surprisingly, the differences in PLT count among groups were statistically significant (FAC vs. CDN vs. TRE = 197.02 ± 84.11 vs. 220.79 ± 59.35 vs. 248.82 ± 106.92, *p* = 0.013), as evidenced by higher blood PLT levels in the TRE group compared to FAC and CDN groups (Figure 2D). Thus, it is worth speculating that *L. rhamnosus* JYLR-127 supplementation in fracture patients with post-surgery constipation may help suppress infection and the inflammatory response, and to some extent control postoperative bleeding tendency. See Supplementary Materials (Figure S1) for inter-group differences of other indicators in Table 2.

### 3.3. L. rhamnosus JYLR-127 Influenced the Composition and Diversity of Gut Microbiota in Fracture Patients with Post-Surgery Constipation

**Figure 3:**
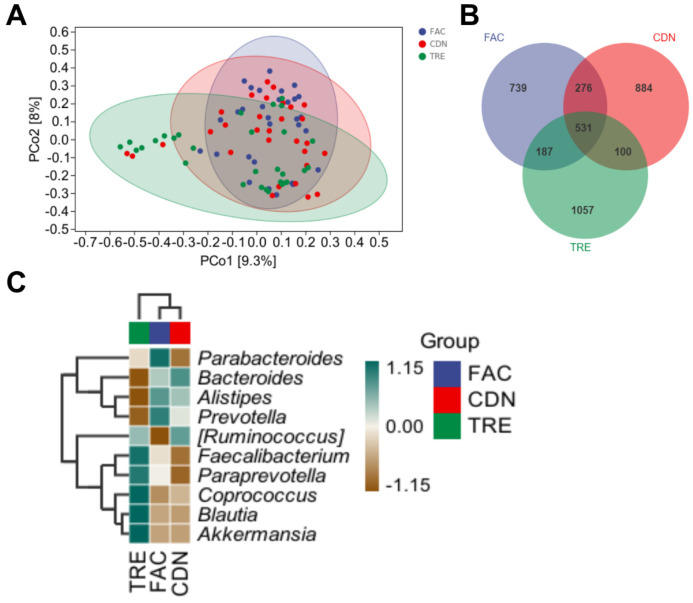
*L. rhamnosus* JYLR-127 transformed the gut microbiota diversity in fracture patients with post-surgery constipation. (**A**) PCoA analysis; (**B**) Venn diagram; (**C**) microbiota composition heatmap. FAC group: N = 30; CDN group: N = 30; TRE group: N = 30, the same as below.

16S rRNA sequencing was performed on fecal samples of FAC, CDN and TRE groups (30 in each) to further obtain multidimensional information on the effect of *L. rhamnosus* JYLR-127 intervention on the gut microbiota of fracture patients with post-surgery constipation. β-diversity, also known as inter-habitat diversity, responds to the dissimilarity of species composition among the three sample groups. The downscaled PCoA model was adopted to simplify analyses, with a more discrete microbiota composition yielded in the TRE group compared to the pre-intervention FAC group and the placebo-controlled CDN group (Figure 3A). In addition, 1733, 1791 and 1875 ASVs were identified in the FAC, CDN and TRE groups, where the percentage of ASVs shared by the three groups was 531/1733 (30.64%), 531/1791 (29.65%) and 531/1875 (28.32%), respectively. The TRE group exhibited a slightly higher abundance of ASVs than the other two groups, highlighting that different interventions may shape specific microbiota structures (Figure 3B). To holistically explore the intestinal microecological characteristics of fracture patients with post-surgery constipation and the alterations in the dominant microbiota after *L. rhamnosus* JYLR-127 administration, a composition heatmap was drawn to identify the dominant bacteria among the FAC, CDN and TRE groups. In Figure 3C, the FAC and CDN groups mainly consisted of *Bacteroides*, *Alistipes* and *Prevotella*. Probiotics, such as *Faecalibacterium*, *Paraprevotella*, *Coprococcus*, *Blautia* and *Akkermansia*, exhibited dominance in the TRE taxa treated with *L. rhamnosus* JYLR-127. As a whole, *L. rhamnosus* JYLR-127 could enrich the composition and hierarchical structure of the gut microbiota in fracture patients with post-surgery constipation to some extent, which is of positive significance for the maintenance of intestinal homeostasis and the amelioration of constipation symptoms.

### 3.4. L. rhamnosus JYLR-127 Regulated the Gut Microbiota Composition in Fracture Patients with Post-Surgery Constipation at Phylum and Genus Levels

**Figure 4:**
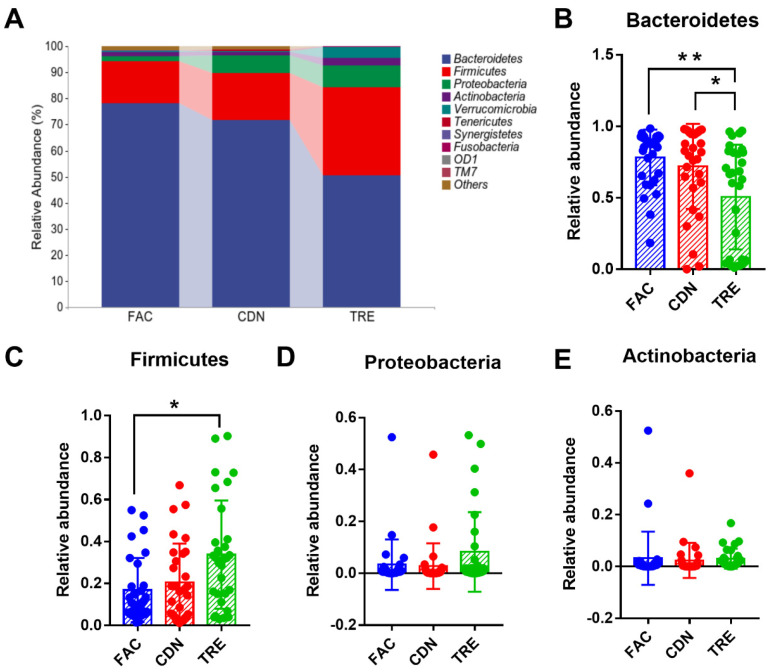
*L. rhamnosus* JYLR-127 affected gut microbiota composition at the phylum level in fracture patients with post-surgery constipation. (**A**) Microbial species composition at the phylum level and the relative abundance of (**B**) Bacteroidetes, (**C**) Firmicutes, (**D**) Proteobacteria, and (**E**) Actinobacteria were shown. * *p* < 0.05, ** *p* < 0.01.

Further, we depicted and visualized the gut microbiome composition and intergroup differences among FAC, CDN and TRE groups at the phylum level. As graphically displayed in Figure 4A, the abundance of Bacteroidetes, Firmicutes, Proteobacteria, and Actinobacteria accounts for the largest proportion of the gut microbiota. Compared to the FAC and CDN groups, the TRE group showed a notable decrease in Bacteroidetes (FAC 78.19% vs. CDN 71.77% vs. TRE 50.51%, *p* < 0.05, Figure 4B) and an increase in Firmicutes, whose difference was statistically significant with the FAC group (FAC 16.09% vs. CDN 17.99% vs. TRE 33.76%, *p* < 0.05, Figure 4C). However, the relative proportions of Proteobacteria and Bacteroidetes were only slightly elevated (FAC 1.96% vs. CDN 6.68% vs. TRE 8.21%, FAC 1.57% vs. CDN 1.39% vs. TRE 3.01%, respectively, Figure 4D,E). Thus, *L. rhamnosus* JYLR-127 could regulate the gut microbiota of fracture patients with post-surgery constipation at the phylum level mainly by increasing the abundance of Firmicutes while decreasing Bacteroidetes.

**Figure 5:**
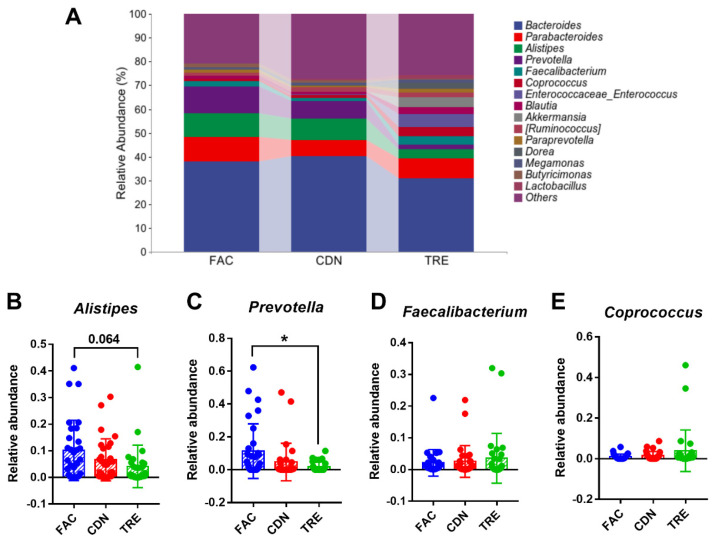
*L. rhamnosus* JYLR-127 altered gut microbiota composition in fracture patients with post-surgery constipation at genus level. (**A**) Microbial species composition at the genus level and the relative abundance of (**B**) *Alistipes*, (**C**) *Prevotella*, (**D**) *Faecalibacterium*, and (**E**) *Coprococcus* were presented, * *p* < 0.05.

At the genus level, the dominance of the *Bacteroides*, *Parabacteroides*, *Alistipes* and *Prevotella* was observed in all three groups in Figure 5A, with a richer and more homogeneous gut microbiota distribution in the TRE group. The relative abundance of conditionally pathogenic bacteria *Alistipes* and conditioned bacteria *Prevotella* in the TRE group was lower than in the FAC and CDN groups (FAC 10.10% vs. CDN 8.98% vs. TRE 4.10%, FAC 11.33% vs. CDN 7.33% vs. TRE 1.75%, respectively, Figure 5D,E). Compared to the FAC group, the reduction in *Prevotella* was statistically significant (*p* < 0.05), *Alistipes* reached the *p*-value threshold (*p* = 0.064), and the abundance of *Bacteroides* (TRE 31.05% vs. FAC 37.96%) and *Parabacteroides* (TRE 8.12% vs. FAC 10.24%) was also reduced (Figure S2A,B). Moreover, whilst not statistically different, an upward trend in the abundance of multiple probiotics like *Faecalibacterium* and *Coprococcus* was seen in the TRE group (FAC 2.13% vs. CDN 1.17% vs. TRE 3.56%, FAC 1.00% vs. CDN 1.43% vs. TRE 3.94%, respectively). Altogether, *L. rhamnosus* JYLR-127 primarily restrained the relative abundance of opportunistic genera, especially *Alistipes* and *Prevotella*, thereby re-establishing healthier gut microbiota for fracture patients with post-surgery constipation symptoms.

### 3.5. L. rhamnosus JYLR-127-Mediated Gut Microbiota Improvement Was Correlated with the Alleviation of Constipation and Infection after Fracture Surgery

**Figure 6:**
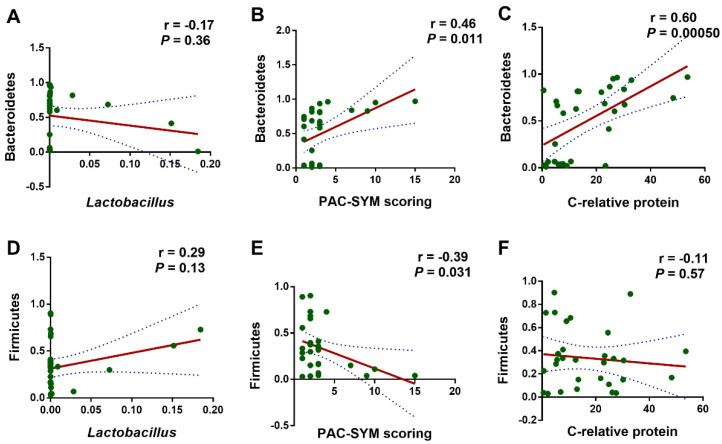
*L. rhamnosus* JYLR-127-mediated gut microbiota improvement was correlated with the alleviated symptoms of constipation and infection after fracture surgery. Pearson correlation analysis was conducted between (**A**) the relative abundance of *Lactobacillus* and Bacteroidetes; (**B**) PAC-SYM scoring and the relative abundance of Bacteroidetes; (**C**) the concentration of C-relative protein and the relative abundance of Bacteroidetes; (**D**) the relative abundance of *Lactobacillus* and Firmicutes; (**E**) PAC-SYM scoring and the relative abundance of Firmicutes; (**F**) the concentration of C-relative protein and the relative abundance of Firmicutes. *p*-Values were obtained by FDR correction.

We further employed Pearson correlation analysis to refine the correlation among *L. rhamnosus* JYLR-127 administration, gut microbiota alteration, and the improvement in symptoms of constipation and infection in fracture patients after surgery. Bacteroidetes and Firmicutes were the phyla with the most significant shifts in the TRE group, rendering them our major concern. Especially for Bacteroidetes, whose relative abundance was positively correlated with PAC-SYM scale scores (indicating constipation symptoms) and serum C-reactive protein concentrations (signaling the level of infection and inflammation in vivo) in fracture patients with post-surgery constipation, the correlations were statistically significant (Figure 6B,C, r = 0.46, *p* = 0.011; r = 0.60, *p* = 0.0005, respectively). Firmicutes showed the opposite, with no significant correlation in the dimension of serum C-reactive protein concentration (Figure 6E,F, r = −0.39, *p* = 0.031; r = −0.11, *p* = 0.57, respectively). The above results suggest a correlation between the reduction in Bacteroidetes and the up-regulation of Firmicutes in gut microbiota after *L. rhamnosus* JYLR-127 intake and the alleviation of constipation as well as the symptoms of the infection. However, the results in Figure 6A,D showed no significant correlation in the abundance of *Lactobacillus* with the Bacteroidetes and Firmicutes (r = −0.17, *p* = 0.36; r = 0.29, *p* = 0.13, respectively), which may be attributed to the limited duration of *L. rhamnosus* JYLR-127 administration, and that the efficacy of *L. rhamnosus* JYLR-127 may also be achieved through modulating the overall microecology of the patients.

## 4. Discussion

Trauma, aging and the ravages of war rank the top three reasons for the prevalence of fractures in recent years [17]. Complications such as constipation and the risk of infection in post-surgery fracture patients hinder the rehabilitation process, posing a burden to clinical practice [18]. The incidence of constipation in hospitalized patients is statistically up to 75% [9], which may be related to post-surgery management, medication, and mental status, thus new adjunctive therapies for intervention and alleviation are urgently needed. The consistent benefits of probiotic preparations in maintaining intestinal homeostasis and physiological health make them our priority [19]. Therefore, in this study, we administrated *L. rhamnosus* JYLR-127 to 100 fracture patients with post-surgery constipation to evaluate its potential in improving gastrointestinal symptoms and anti-inflammatory responses.

It is worth emphasizing that despite the proven efficacy of *L. rhamnosus* in the treatment of chronic constipation [20], fracture patients with a history of chronic constipation were excluded at the outset of recruitment to ascertain consistency and comparability at baseline. In addition, the application of antibiotics was not strictly limited, as it is necessary for fracture patients to take antibiotics to prevent bacterial invasion and infection, even septicemia [21]. Indeed, the intestinal microecology of the post-fracture patients in this study was fragile and relatively monotonous due to antibiotic usage, trauma-induced immune disorder and postoperative dietary restriction. The administration of *L. rhamnosus* JYLR-127 made multiple bacteria in Firmicutes stand out as dominant and inhibited the colonization of conditionally pathogenic bacteria, which proved that *L. rhamnosus* JYLR-127 could to some extent circumvent the effect of antibiotics and play a positive role. Consistent with our results, there are studies suggesting that long-term supplementation with *L. rhamnosus* GG benefits the intestinal microbiota composition, protects against penicillin-associated microbiota disruption, and provides long-lasting protection of the intestinal barrier function [22].

Undoubtedly, oral supplementation with *L. rhamnosus* JYLR-127 (2 times/day for one week) has been shown to ameliorate the symptoms of constipation after fracture surgery. The increased defecation frequency in the TRE group indicated an improvement in bowel motility, the median Bristol score symbolized the normalization of fecal traits, and the decreased PAC-SYM scale scores parallelly represented a general restoration of gastrointestinal symptoms in patients [23,24]. Mechanistically, the above results were mainly attributed to the reconstruction of the disrupted gut microbiota and the modification of the intestinal microecology by probiotics intake orally. Although no obvious difference was found in global assessments of species diversity before and after *L. rhamnosus* JYLR-127 intervention as well as the placebo control, the rise in Firmicutes and the decline in Bacteroidetes were statistically significant. In the species composition heatmap, the dominant genera of TRE samples were concentrated in certain probiotics, which differed significantly from the FAC and CDN groups. It can be surmised that one week after the intervention the gut microbiota of patients was still in the process of rebuilding, although dominant species emerged and took the lead, the reconstruction of many microscopic bacteria was not yet complete. On the basis of Pearson correlation analysis, we focused on the increased Firmicutes and decreased Bacteroides at the phylum level, especially the latter, to which *Alistipes* and *Prevotella*, observed with significant differences at the genus level, belong. Together, these two phyla constitute more than 90% of the gut microbiota, with the Firmicutes/Bacteroidetes (F/B) ratio being a universal indicator in evaluating the dynamic balance of the intestinal tract [25]. Firmicutes contain predominately butyrate-yielding bacteria, which work in maintaining the integrity of the intestinal barrier and participating in the digestion, absorption and metabolic transport of nutrients [26]. At the genus level, *Faecalibacterium* and *Coprococcus* are both biomarkers available to evaluate the health status of the human gastrointestinal tract [27,28]. Bacteroidetes, on the other hand, is more comprehensive and active in functional effects and, due to the presence of wide ranges of pathogenic bacteria, mediates intestinal immunity while also being prone to causing dyspepsia and homeostatic imbalances under unfavorable conditions [29]. The rebound of the F/B ratio reflects the normalization of gut microbiota, which may be one of the targets of *L. rhamnosus* JYLR-127 intervention in improving the post-surgery constipation symptoms of fracture patients.

The suppression of inflammation and infection in fracture patients with post-surgery constipation by oral administration of *L. rhamnosus* JYLR-127 was also the concern explored in this study. Studies have shown a positive correlation between C-reactive protein levels with the degree of infection and injury, which is necessary for evaluating the activities of acute inflammation and surgical trauma, as well as the efficacy of medications [30]. Although the only observed significance lay in the decrease in C-reactive protein in the probiotic intervention group compared to the placebo control, the anti-inflammatory and anti-infective effects of *L. rhamnosus* JYLR-127 could be reflected. *Alistipes* and *Prevotella* were considered pro-inflammatory bacteria, with mounting inflammatory diseases related, such as irritable bowel, colorectal cancer, and cirrhosis [31,32]. One controversy is *Prevotella*, whose nature as an opportunistic pathogenic requires us to invest more time in investigating its role in inflammation inhibition in post-fracture constipation patients. PLT has been adopted to determine the hypo or hypercoagulable state of the blood and thus target medications to prevent bleeding tendencies or thrombosis in post-fracture patients [33]. Interestingly, PLT, which was originally only a minor indicator that we tested in routine blood tests, showed unexpected statistical differences between the TRE and CDN groups, which led us to consider the possibility of *L. rhamnosus* JYLR-127 for coagulation improvement in post-fracture constipation patients with low PLT counts.

Inevitably, there are some shortcomings in this study. Due to the limited number of fracture patients with post-surgery constipation recruited, statistical analysis may be subject to error and bias. Perhaps the rigorous inclusion of participants and strict management of trial protocol may partially compensate for this drawback by improving patient compliance and the reliability of the results. Given that most constipation induced by physical blows is mild [34], we considered it ideal to take short-term probiotic intervention. Also, patients undergoing fracture rehabilitation are usually discharged from the hospital within an average of 2–3 weeks postoperatively [35], while continuing interventions and follow-up after discharge are difficult and compliance cannot be guaranteed either. This means that from the determination of post-fracture constipation to the completion of the intervention, efficacy validation needs to be conducted. Thus, the experiment duration was controlled to one week, which could explain most of the negative results, but the long-term effect of *L. rhamnosus* JYLR-127 on post-fracture constipation could not be captured. In addition, the unmatched samples pre- and post-intervention may influence the validity of the results to some extent, especially on the microbial data. Although the alleviation of constipation and inflammation has been observed simultaneously, as well as the correlation between gut microbiota remodeling and symptom improvement, it was not yet possible to link both organically. Therefore, operations such as multi-omics analysis based on the metabolites of the organism and gut microbiota, and the establishment of a post-fracture constipation rat model might be applied in the follow-up to clarify the potential mechanism of *L. rhamnosus* JYLR-127 on post-fracture constipation.

## 5. Conclusions

In conclusion, our study identified more vulnerable and susceptible intestinal microecology with elevated systemic inflammatory response, infection risk and bleeding tendency in fracture patients with post-surgery constipation. While *L. rhamnosus* JYLR-127 intervention significantly increased the relative abundance of Firmicutes and reduced Bacteroidetes, especially *Alistipes* and *Prevotella*, which helped rebuild the impaired gut microbiota, promote defecation function, improve constipation symptoms, and at the same time resist inflammation and infection. These results confirmed the benefit of *L. rhamnosus* JYLR-127 administration in eliminating the complications of fracture patients with post-surgery constipation, which was anticipated to enhance prognosis and accelerate fracture rehabilitation.

## Figures and Tables

**Table 1:** Baseline characteristics of the subjects.

Characteristics		FAC Group	CDN Group	TRE Group	*p*-Value
Number (N)		50	47	49	/
Age (years)		54.00 ± 11.08	53.85 ± 11.46	55.06 ± 10.14	0.837
Gender (male/female)		29/21	28/19	29/20	/
Fracture type	limbs	16 (32%)	14 (30%)	14 (29%)	/
	spinal	29 (58%)	30 (64%)	30 (61%)	/
	multiple	5 (10%)	3 (6%)	5 (10%)	/
Body Mass Index (BMI)		22.30 ± 1.62	22.20 ± 1.61	22.04 ± 1.57	0.979

Statistics were presented as mean ± standard deviation (SD).

**Table 2:** The differences in fecal and blood indexes among groups.

Indicators	FAC Group (N = 50)	CDN Group (N = 47)	TRE Group (N = 49)	*p*-Value
Defecation frequency	2.24 ± 0.72	2.06 ± 0.70	3.04 ± 1.54	<0.001 (***)
Bristol scoring	3.16 ± 0.99	2.99 ± 0.82	3.63 ± 0.95	0.003 (**)
PAC-SYM scoring	4.72 ± 2.78	4.81 ± 2.47	3.28 ± 2.62	0.007 (**)
Rectal symptom	2.74 ± 1.52	3.09 ± 1.52	2.39 ± 1.59	0.090
Abdominal symptom	1.04 ± 0.81	1.13 ± 0.82	0.41 ± 0.54	<0.001 (***)
Fecal symptom	0.94 ± 1.02	1.02 ± 1.05	0.49 ± 0.92	0.020 (*)
C-reactive protein (mg/L)	31.13 ± 41.58	24.45 ± 25.13	15.42 ± 13.02	0.030 (*)
White blood cell (10^9^/L)	8.03 ± 3.33	9.27 ± 3.31	7.81 ± 2.88	0.056
Red blood cell (10^12^/L)	4.29 ± 0.75	4.07 ± 0.80	4.13 ± 0.75	0.347
Platelet (10^9^/L)	197.02 ± 84.11	220.79 ± 59.35	248.82 ± 106.92	0.013 (*)

Statistics were presented as mean ± standard deviation (SD), * *p* < 0.05, ** *p* < 0.01, *** *p* < 0.001. The unit of defecation frequency was set to times per week. The unit of Bristol and PAC-SYM scoring, rectal, abdominal and fecal symptoms were set to scores.

## Data Availability

Data related to the microbiological samples of participants have been uploaded to the SRA database at BioProject PRJNA1093356. Other raw data are available from the corresponding authors upon reasonable request.

## Supplementary Materials

The following supporting information can be downloaded at: https://www.mdpi.com/article/10.3390/nu16101505/s1, Figure S1: *L. rhamnosus* JYLR-127 improved post-surgery constipation symptoms and mediated anti-inflammation and anti-infection in the organism. The comparison of other indicators including (A) Defecation frequency, (B) Rectal symptom, (C) Abdominal symptom, (D) Fecal symptom, (E) White blood cell (WBC) and (F) Red blood cell (RBC) was visualized here. FAC: pre-intervention group (N = 50); CDN: placebo control group (N = 47); TRE: *L. rhamnosus* JYLR-127 group (N = 49). * *p* < 0.05, ** *p* < 0.01; Figure S2: *L. rhamnosus* JYLR-127 altered gut microbiota composition in fracture patients with post-surgery constipation at genus level. The relative abundance of (A) *Bacteroides* and (B) *Parabacteroides* were presented. FAC group: N = 30; CDN group: N = 30; TRE group: N = 30, * *p* < 0.05.

## References

[1] Saul D., Khosla S.A.-O. (2022). Fracture Healing in the Setting of Endocrine Diseases, Aging, and Cellular Senescence. Endocr. Rev..

[2] GBD 2019 Fracture Collaborators (2021). Global, regional, and national burden of bone fractures in 204 countries and territories, 1990–2019: A systematic analysis from the Global Burden of Disease Study 2019. Lancet Healthy Longev..

[3] Caricato A.A.-O.X., Russo G., Biasucci D.G., Annetta M.G. (2017). Fat embolism syndrome. Intensive Care Med..

[4] Dudareva M., Hotchen A.J., Ferguson J., Hodgson S., Scarborough M., Atkins B.L., McNally M.A. (2019). The microbiology of chronic osteomyelitis: Changes over ten years. J. Infect..

[5] Handoll H.H., Elliott J. (2015). Rehabilitation for distal radial fractures in adults. Cochrane Database Syst. Rev..

[6] Viberg B., Erlandsen Claville L.U., Andersen L.R., Fredholm L., Dall-Hansen D., Grejsen H. (2022). Standardized, Coordinated Care in Nursing Homes Lowers Rehospitalization after Hip Fracture. J. Am. Med. Dir. Assoc..

[7] Barberio B., Judge C., Savarino E.V., Ford A.C. (2021). Global prevalence of functional constipation according to the Rome criteria: A systematic review and meta-analysis. Lancet Gastroenterol. Hepatol..

[8] Reid I.R., Mason B., Horne A., Ames R., Reid H.E., Bava U., Bolland M.J., Gamble G.D. (2006). Randomized controlled trial of calcium in healthy older women. Am. J. Med..

[9] Jing D., Jia L. (2019). Assessment of patients’ psychological state and self-efficacy associated with postoperative constipation after thoracolumbar fracture surgery. J. Int. Med. Res..

[10] Liang Z.Q., Chen S.X. (2020). Effects of Jiawei Tiaowei Chengqi Decoction on gastrointestinal function recovery and intestinal flora in elderly patients after hip fracture surgery. Chin. Foreign Med. Res..

[11] Yin H., Wang G., Wang J., Ma Y., Wu M., Qiu S., Su Q. (2021). Prevalence and Risk Factor Analysis of Constipation after Thoracolumbar Vertebral Compression Fractures. Int. J. Gen. Med..

[12] Redman M.G., Ward E.J., Phillips R.S. (2014). The efficacy and safety of probiotics in people with cancer: A systematic review. Ann. Oncol..

[13] Suez J., Zmora N., Segal E., Elinav E. (2019). The pros, cons, and many unknowns of probiotics. Nat. Med..

[14] Goldstein E.J., Tyrrell K.L., Citron D.M. (2015). Lactobacillus species: Taxonomic complexity and controversial susceptibilities. Clin. Infect. Dis..

[15] Lai H., Li Y., He Y., Chen F., Mi B., Li J., Xie J., Ma G., Yang J., Xu K. (2023). Effects of dietary fibers or probiotics on functional constipation symptoms and roles of gut microbiota: A double-blinded randomized placebo trial. Gut Microbes.

[16] Chen J.F., Zhuang Y., Jin S.B., Zhang S.L., Yang W.W. (2021). Probiotic *Lactobacillus rhamnosus* GG (LGG) restores intestinal dysbacteriosis to alleviate upregulated inflammatory cytokines triggered by femoral diaphyseal fracture in adolescent rodent model. Eur. Rev. Med. Pharmacol. Sci..

[17] Tomás C.C., Oliveira E., Sousa D., Uba-Chupel M., Furtado G., Rocha C., Teixeira A., Ferreira P., Alves C., Gisin S. (2016). Proceedings of the 3rd IPLeiria’s International Health Congress: Leiria, Portugal. 6–7 May 2016. BMC Health Serv. Res..

[18] Liu R., Chao A., Wang K., Wu J. (2018). Incidence and risk factors of medical complications and direct medical costs after osteoporotic fracture among patients in China. Arch. Osteoporos..

[19] van Baarlen P., Wells J.M., Kleerebezem M. (2013). Regulation of intestinal homeostasis and immunity with probiotic lactobacilli. Trends Immunol..

[20] Currò D., Ianiro G., Pecere S., Bibbò S., Cammarota G. (2017). Probiotics, fibre and herbal medicinal products for functional and inflammatory bowel disorders. Br. J. Pharmacol..

[21] Moriarty T.F., Metsemakers W.J., Morgenstern M., Hofstee M.I., Vallejo Diaz A., Cassat J.E., Wildemann B., Depypere M., Schwarz E.M., Richards R.G. (2022). Fracture-related infection. Nat. Rev. Dis. Primers.

[22] Duysburgh C., Van den Abbeele P., Morera M., Marzorati M. (2021). *Lacticaseibacillus rhamnosus* GG and Saccharomyces cerevisiae boulardii supplementation exert protective effects on human gut microbiome following antibiotic administration in vitro. Benef. Microbes.

[23] Yang X., Wang J., Cheng J., Zhang D., Huang K., Zhang Y., Li X., Zhao Y., Zhao L., Xu D. (2024). Relationship between sheep feces scores and gastrointestinal microorganisms and their effects on growth traits and blood indicators. Front. Microbiol..

[24] Ibarra A., Latreille-Barbier M., Donazzolo Y., Pelletier X., Ouwehand A.C. (2018). Effects of 28-day Bifidobacterium animalis subsp. lactis HN019 supplementation on colonic transit time and gastrointestinal symptoms in adults with functional constipation: A double-blind, randomized, placebo-controlled, and dose-ranging trial. Gut Microbes.

[25] Stojanov S., Berlec A., Štrukelj B. (2020). The Influence of Probiotics on the Firmicutes/Bacteroidetes Ratio in the Treatment of Obesity and Inflammatory Bowel disease. Microorganisms.

[26] Stoeva M.K., Garcia-So J., Justice N., Myers J., Tyagi S., Nemchek M., McMurdie P.J., Kolterman O., Eid J. (2021). Butyrate-producing human gut symbiont, Clostridium butyricum, and its role in health and disease. Gut Microbes.

[27] Martín R., Rios-Covian D., Huillet E., Auger S., Khazaal S., Bermúdez-Humarán L.G., Sokol H., Chatel J.M., Langella P. (2023). Faecalibacterium: A bacterial genus with promising human health applications. FEMS Microbiol. Rev..

[28] Yang R., Shan S., Shi J., Li H., An N., Li S., Cui K., Guo H., Li Z. (2023). Coprococcus eutactus, a Potent Probiotic, Alleviates Colitis via Acetate-Mediated IgA Response and Microbiota Restoration. J. Agric. Food Chem..

[29] Zafar H., Saier M.H. (2021). Gut Bacteroides species in health and disease. Gut Microbes.

[30] Póvoa P., Coelho L., Dal-Pizzol F., Ferrer R., Huttner A., Conway Morris A., Nobre V., Ramirez P., Rouze A., Salluh J. (2023). How to use biomarkers of infection or sepsis at the bedside: Guide to clinicians. Intensive Care Med..

[31] Requena T., Martínez-Cuesta M.C., Peláez C. (2018). Diet and microbiota linked in health and disease. Food Funct..

[32] Parker B.J., Wearsch P.A., Veloo A.C.M., Rodriguez-Palacios A. (2020). The Genus Alistipes: Gut Bacteria with Emerging Implications to Inflammation, Cancer, and Mental Health. Front. Immunol..

[33] Zhang Z., Li Z., Li J., Liu L. (2018). Effects of Natural Hirudin and Low Molecular Weight Heparin in Preventing Deep Venous Thrombosis in Aged Patients with Intertrochanteric Fracture. Sci. Rep..

[34] Vandeputte D., Falony G., Vieira-Silva S., Wang J., Sailer M., Theis S., Verbeke K., Raes J. (2017). Prebiotic inulin-type fructans induce specific changes in the human gut microbiota. Gut.

[35] Wong B.L.L., Chan Y.H., O’Neill G.K., Murphy D., Merchant R.A. (2023). Frailty, length of stay and cost in hip fracture patients. Osteoporos. Int..

