# Supplementary material for: Improvement of Post-Surgery Constipation in Patients with Fractures by Lactobacillus rhamnosus JYLR-127: A Single-Blind Randomized Controlled Trial
Source: Nutrients. 2024 May 16;16(10):1505. doi: 10.3390/nu16101505 (PMC11123980; doi:10.3390/nu16101505)
Supplement: Supplementary file 1 [file nutrients-16-01505-s001.zip › nutrients-2983745-supplementary.pdf]

Supplementary Materials

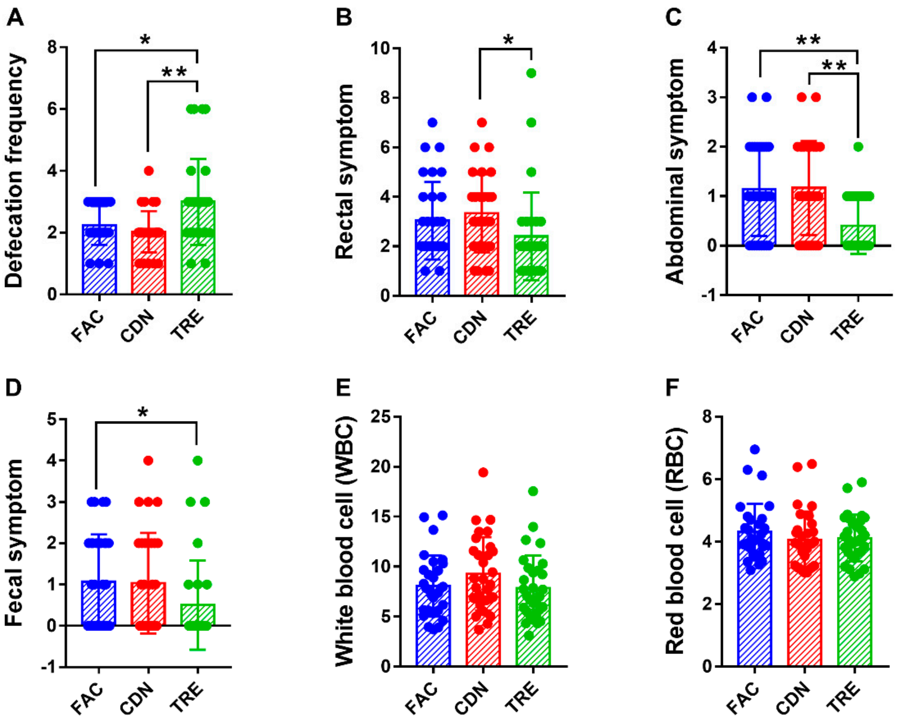

Figure S1. ***L. rhamnosus*** **JYLR-127 improved post-surgery constipation symptoms and mediated anti-inflammation and anti-infection in the organism.** The comparison of other indicators including (**A**) Defecation frequency, (**B**) Rectal symptom, (**C**) Abdominal symptom, (**D**) Fecal symptom, (**E**) White blood cell (WBC) and (**F**) Red blood cell (RBC) was visualized here. FAC: pre-intervention group (N = 50); CDN: placebo control group (N = 47); TRE: *L. rhamnosus* JYLR-127 group (N = 49). * $p < 0.05$, ** $p < 0.01$.

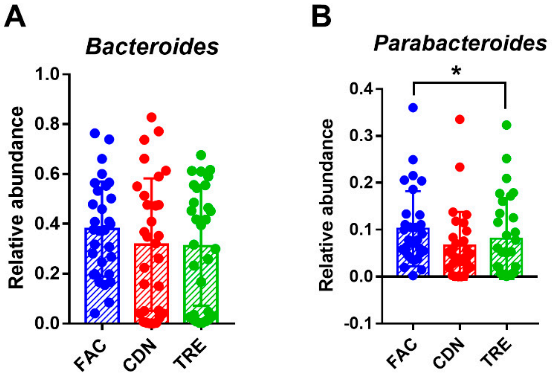

Figure S2. ***L. rhamnosus*** **JYLR-127 altered gut microbiota composition in fracture patients with post-surgery constipation at genus level.** The relative abundance of (**A**) *Bacteroides* and (**B**) *Parabacteroides* were presented. FAC group: N = 30; CDN group: N = 30; TRE group: N = 30, * $p < 0.05$.
